# Magnetic sphincter augmentation (MSA) in patients with hiatal hernia: clinical outcome and patterns of recurrence

**DOI:** 10.1007/s00464-019-06950-4

**Published:** 2019-07-08

**Authors:** Shahin Ayazi, Nobel Chowdhury, Ali H. Zaidi, Kristy Chovanec, Yoshihiro Komatsu, Ashten N. Omstead, Ping Zheng, Toshitaka Hoppo, Blair A. Jobe

**Affiliations:** Esophageal and Lung Institute, Allegheny Health Network, 4815 Liberty Avenue, Suite 439, Pittsburgh, PA 15224 USA

**Keywords:** Gastroesophageal reflux disease (GERD), Hiatal hernia, Magnetic sphincter augmentation

## Abstract

**Introduction:**

Magnetic sphincter augmentation (MSA) is an effective treatment for patients with gastroesophageal reflux disease. In early studies, patients with a hiatal hernia (HH) ≥ 3 cm were excluded from consideration for implantation and initially the FDA considered its use as “precautionary” in this context. This early approach has led to an attitude of hesitance among some surgeons to offer this therapy to patients with HH. This study was designed to evaluate the impact of HH status on the outcome of MSA and to report the rate of HH recurrence after MSA.

**Methods and procedures:**

This is a retrospective review of prospectively collected data of patients who underwent MSA between June 2013 and August 2017. Baseline clinical and objective data were collected. Patients were divided into four groups based on HH status: no HH, small HH (< 3 cm), large HH (≥ 3 cm), and paraesophageal hernia (PEH). Patient satisfaction, GERD–HRQL and RSI data, freedom from PPI, need for postoperative dilation, length of hospitalization, 90-day readmission rate, need for device removal, and HH recurrence was compared between groups.

**Results:**

There were 350 patients [60% female, mean (SD) age: 53.5 (13.8)] who underwent MSA. There were 65 (18.6%) with no HH, 205 (58.6%) with small HH (< 3 cm), 58 (16.6%) with large HH (≥ 3 cm) and 22 (6.2%) with PEH. At a mean follow-up of 13.6 (10.4) months, the rate of outcome satisfaction was similar between the groups (86%, 87.9%, 92.2% and 93.8%, *p* = 0.72). This was also true for GERD–HRQL total score clinical improvement (79.1%, 77.8%, 82% and 87.5%, *p *= 0.77). The rate of postoperative dysphagia (*p *= 0.33) and freedom from PPIs (*p *= 0.96) were similar among the four groups. Duration of hospitalization was higher among those with a large HH or PEH, and only PEH patients had a higher 90-day readmission rate (*p *= 0.0004). There was no difference between the need for dilation among groups (*p *= 0.13). The need for device removal (5% overall) was similar between the four groups (*p *= 0.28). HH recurrence was 10% in all groups combined, and only 7 of 240 (2.9%) patients required reoperation; the majority of these patients underwent a minimal dissection approach (no hernia repair) at the index operation. The incidence of recurrent HH increased in direct correlation with the preoperative HH size (0%, 10.1%, 16.6 and 20%, *p *= 0.032).

**Conclusion:**

In the largest series of MSA implantation, we demonstrate that the excellent outcomes and high degree of satisfaction after MSA are independent of the presence or size of HH. Despite higher rates of hernia recurrence in large HH and PEH patients, the rates of postoperative endoscopic intervention, and device removal is similar to those with no or small HH. The minimal dissection approach to MSA should be abandoned.

Gastroesophageal reflux disease (GERD) is the most common foregut disease that affects about 10% of the western population [1–3]. In this chronic disease, both the crural contribution and intrinsic barrier function of the lower esophageal sphincter (LES) fails and allows reflux of abnormal amounts of gastric juice into the esophagus. The two main treatment options for patients with GERD are long-term acid suppression therapy with proton-pump inhibitors (PPI) or laparoscopic fundoplication. Medical acid suppression therapy is an effective first-line therapy in most patients. However, nearly 40% of patients experience breakthrough symptoms [4, 5]. In addition, there are potential risks associated with PPIs including B12 vitamin deficiency, Clostridium difficile infection, community-acquired pneumonia, and osteoporosis [6–8]. Other consequences of prolonged PPI therapy include hypergastrinemia, enterochromaffin-like cell hyperplasia, and parietal cell hypertrophy, leading to rebound acid hypersecretion [9, 10].

Laparoscopic Nissen fundoplication is the surgical treatment option offered to patients whose condition has failed to respond to medical therapy or who desire to be free from dependence on medical therapy. However, this operation is underused due to the fears of long-term side effects such as gas bloat, inability to belch or vomit, and anatomic failure of the repair. The limitations of pharmacologic therapy and fundoplication leave many patients and clinicians in the difficult position to either tolerate a lifetime of drug dependence with incomplete symptom relief or to undergo a complex surgical procedure that is has been difficult to disseminate on a large-scale, and may have considerable side effects.

Magnetic sphincter augmentation was developed to address the existing ‘therapy gap’ through a laparoscopic procedure, that does not alter gastric anatomy, augments the physiologic barrier to reflux, and is reversible. This procedure was designed to be a technically straightforward and highly reproducible outpatient procedure that centers on the implantation of a device. Because of this, multiple centers across the United States have reported a high degree of success with remarkably consistent clinical outcomes [11–15].

In early studies, patients with a hiatal hernia (HH) ≥ 3 cm were excluded from consideration for implantation and initially the FDA considered its use as “precautionary” in this context [16]. This early approach has led to an attitude of hesitance among some surgeons to offer this therapy to patients with HH, and a minimal dissection approach (no mediastinal dissection or cruralplasty) was recommended. Recent studies have demonstrated encouraging results in the use of MSA in patients with a larger size hiatal hernia [17, 18]. However, there remains a paucity of data on the overall impact of hiatal hernia on the outcome of MSA. This study was designed to compare the outcome of MSA across the spectrum of hiatal hernias commonly encountered in the care of patients with GERD and to review the pattern of hiatal hernia recurrence.

## Methods

### Study population

This is a retrospective review of prospectively collected data of patients who underwent MSA at Allegheny Health Network hospitals (Pittsburgh, PA) between June 2013 and August 2017. Approval was obtained from the Allegheny Health Network Institutional Review Board (IRB 2018-161) prior to the start of the study.

Inclusion criteria were symptomatic GERD patients 18 years or older with persistent GERD or laryngopharyngeal reflux symptoms despite maximal antisecretory therapy and objective evidence of reflux disease based on increased esophageal acid exposure on pH monitoring or a positive impedance-pH based on previously described criteria [19–21]. Patients with a previous history of esophageal or gastric surgery, gross anatomic abnormalities such as esophageal stricture, significant esophageal dysmotility or a known allergy to titanium were not included in this study.

### Preoperative assessment

All patients completed a detailed clinical evaluation with a focus on their foregut symptoms and acid suppression medication use, and completed the Gastroesophageal Reflux Disease–Health Related Quality of Life (GERD–HRQL) and Reflux Symptom Index questionnaires [22, 23] while taking their usual dosing of antisecretory medication. The GERD–HRQL assesses GERD symptoms and patient satisfaction using a 0 to 5 rating scale. It is composed of ten questions relating to the severity of heartburn, regurgitation dysphagia, odynophagia, and bloating. The total GERD–HRQL score is calculated by summing the responses to 10 questions with scores ranging from 0 to 50 [22]. Similarly, the RSI is a validated and reproducible nine-item instrument (each item with a 0 to 5 rating scale) used in assessment of laryngopharyngeal reflux (LPR) symptom severity with a score > 13 being abnormal. Patients completed an objective foregut evaluation prior to consideration for surgery. The routine preoperative objective assessment included the following tests:

1. Esophagogastroduodenoscopy (EGD) with biopsy: to assess the presence of esophagitis, Barrett’s esophagus and the presence and size of a hiatal hernia. The size of HH was recorded in centimeters based on the distance from the gastroesophageal junction to the crural impression. Patients were divided into 4 groups based on HH status: no HH, small HH (< 3 cm), large HH (≥ 3 cm), and paraesophageal hernia (PEH). Small and large HH (Type I) was defined as axial displacement of the gastroesophageal junction (GEJ) and proximal stomach into the chest with all herniated stomach being distal to the GEJ. Paraesophageal hernia (Type III) was defined when both the GEJ and stomach were located intrathoracically with a portion or entire herniated stomach located proximal to the GEJ or in the presence of organoaxial volvulus (“upside down stomach”).
2. High-resolution impedance manometry (HRIM): this test was performed using high-resolution manometry (4.2-mm diameter; Medtronic Inc., MN), equipped with 36 pressure transducers (1 cm apart) to assess the esophageal body peristalsis (organization and pressure) and upper and lower esophageal sphincter pressure, position and length as previously described [24].
3. Esophageal pH or impedance-pH monitoring: these tests were performed selectively using either Bravo pH monitoring (Medtronics, Shoreview, MN, USA) or multichannel intraluminal impedance (MII) pH monitoring (Sandhill Scientific Inc, Highlands Ranch CO) [21, 25]. Prior to pH testing proton pump inhibitors were discontinued for 10 days. A DeMeester score > 14.7 was considered as abnormal distal esophageal acid exposure. Impedance-pH testing was used in patients with predominate symptoms of laryngopharyngeal reflux with or without typical reflux symptoms using previously described criteria [21].
4. Videoesophagram (VEG): this imaging study was done to evaluate gross pharyngeal and esophageal motility, and to further delineate the anatomy and assess for any potential mass or mucosal lesions, diverticulum, and to evaluate hiatal hernia and esophageal stricture or scarring.

### Postoperative and outcome assessment

Subjective postoperative outcomes were evaluated at routine visits at 2 weeks, 6 weeks, 3 months, and then yearly after surgery. Patients were assessed for resolution of their reflux symptoms, use of antisecretory medications, and procedure-related complications. Length of hospital stay, need for readmission within 90 days after surgery, and need for postoperative dilation and device removal were also recorded. Patients were asked to complete GERD-HRQL and RSI questionnaires at their 6 month and yearly visits. A 50% improvement in the total GERD–HRQL score compared with the baseline on antisecretory therapy was considered clinically significant in this study. Using the RSI, late postoperative dysphagia was defined as a postoperative dysphagia score ≥ 3 on the ‘difficulty swallowing’ item at ≥ 8 weeks after MSA.

At 1-year following MSA, patients were approached for objective foregut evaluation using the same tests employed in the preoperative evaluation. Recurrence of HH was determined based on follow-up upper endoscopy at 1-year follow-up or if the patient presented with suggestive symptoms prior to that time [26].

A recurrence was considered present if the GEJ was found to be proximal to the crural impressions on either anterograde or retroflexion endoscopic view.

### Device and surgical procedure

The LINX device (Ethicon, Johnson & Johnson, Shoreview, MN) consists of a series of titanium beads with magnetic cores hermetically sealed inside. The beads are interlinked with independent titanium wires to form a flexible and expandable ring with a ‘Roman arch’ configuration. Each bead can move independently of the adjacent beads, creating a dynamic implant that mimics the physiological movement of the esophagus without limiting its range of motion. The device is manufactured in different sizes, from 13 to 17 beads, and is capable of nearly doubling its diameter when all beads are separated.

This procedure is performed laparoscopically and consists of complete posterior mediastinal esophageal mobilization with restoration of intraabdominal esophageal length (≥ 3 cm), interrupted posterior crural closure (without pledgets or mesh) and device placement at the level of the GEJ with the posterior vagus nerve trunk located on the outside of the magnetic ring.

Early in our experience, a ‘minimal dissection’ technique was used in patients with little to no HH; this approach does not include mediastinal esophageal dissection, the phrenoesophageal ligament is left intact, and there is no crural closure. A small window is created within the retroesophageal space, and the device is placed around the GEJ.

**Fig. 1:**
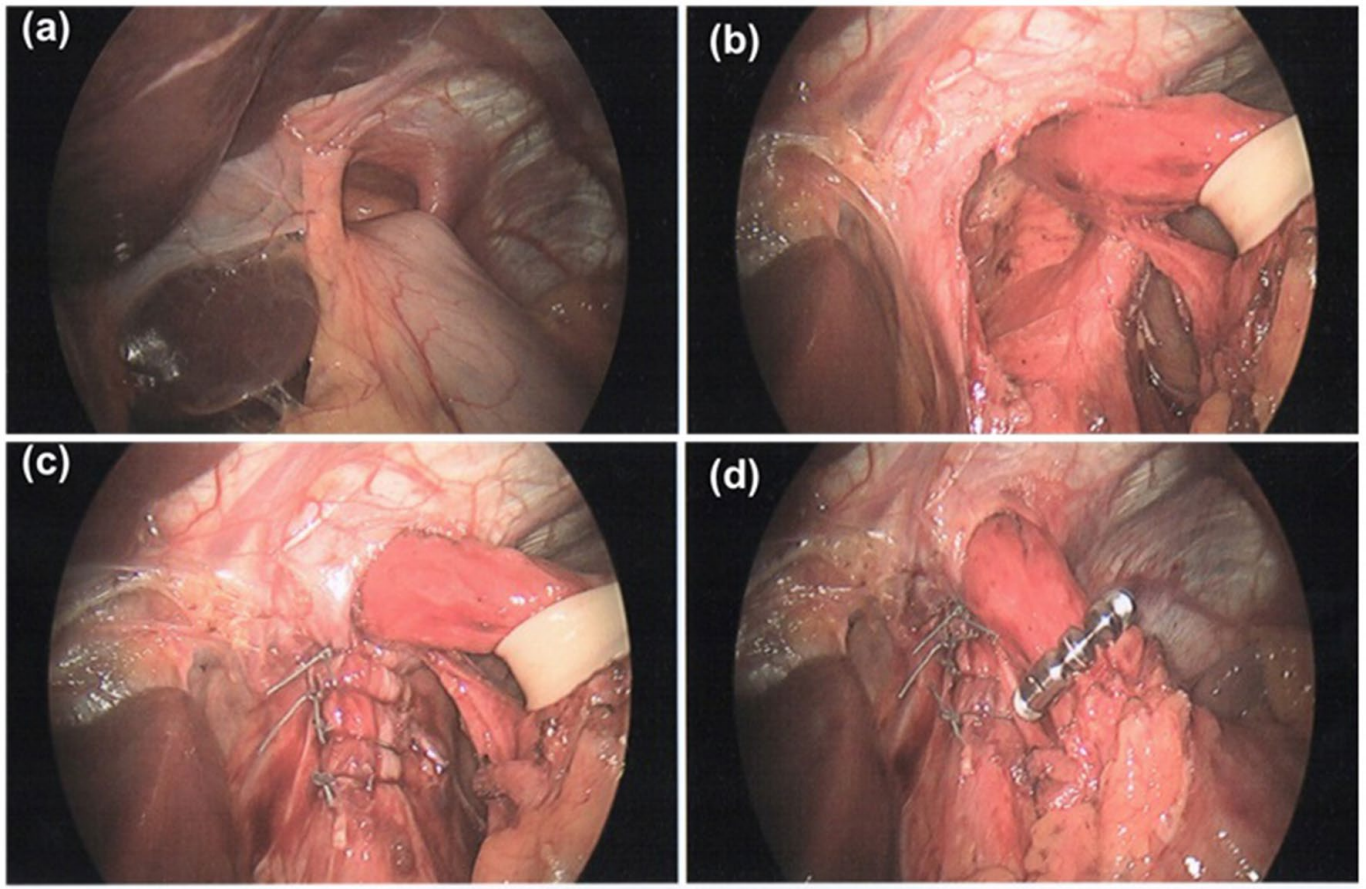
Steps of hernia repair and magnetic sphincter augmentation in a patient with large PEH

A sizing procedure, which assesses esophageal circumference, is performed prior to selecting the size of device. This approach is used in all patients regardless of whether there is a preoperative diagnosis of hiatal hernia. Many patients have transverse widening of the hiatal opening with minimal axial displacement and our approach is focused on restoring the crural contribution of the antireflux barrier during MSA placement. Intraoperative esophagogastroscopy is performed in order to assist in identifying the anatomic GEJ and to assess device position (Fig. 1).

### Statistical analysis

Values are expressed as either mean with standard deviation (SD) or median with interquartile range (IQR) when appropriate. Statistical analysis was performed by means of nonparametric Mann–Whitney *U* test, Wilcoxon signed-rank test, and Person’s Chi-square test when appropriate. A *p* value < 0.05 was considered significant. Statistical analysis was performed using SAS software (SAS Institute Inc., Cary, N.C.).

Patients were divided into four groups based on HH status: no HH, small HH (< 3 cm), large HH (≥ 3 cm), and paraesophageal hernia (PEH). Patient satisfaction, GERD–HRQL data, freedom from PPI, need for postoperative dilation, length of hospitalization, 90-day readmission rate, the need for device removal, and HH recurrence were compared between groups.

## Results

There were 350 patients who underwent magnetic sphincter augmentation during the study period and were included in this analysis. Patients were mostly in the fifth decade of life and there were more women than men (Table 1).

**Table 1:** Baseline demographic and clinical characteristics

Characteristics	*N* (%)
Age (year)	
Mean (SD)	53.5 (13.8)
Gender	
Male	141 (40.3%)
Female	209 (59.7%)
BMI	
Mean (SD)	29.2 (4.7)
PPI use	251 (89%)
DeMeester score	
Mean (SD)	32.6 (27.5)
*N* (%) with abnormal score (≥ 14.72)	144 (74.6%)
Hiatal hernia	
Yes	285 (81.4%)
No	65 (18.6%)
Size and type of hernia	
Small (≤ 3 cm)	205 (71.9%)
Large (≥ 3 cm)	58 (20.4%)
PEH	22 (7.7%)

### Preoperative status of hiatal hernia and outcome

A total of 285 patients were found to have a hiatal hernia on their preoperative endoscopy. Of these, 205 (71.9%) had a small hernia, 58 (20.4%) had a large hernia and 22 (7.7%) had a paraesophageal hernia. Of the 65 patients with no evidence of hernia on preoperative evaluation, 28 (30%) were found to have transverse crural separation and a small “dimple” within the phrenoesophageal ligament anteriorly as viewed laparoscopically. These patients remained classified within the no hernia group and 38 patients underwent the minimal dissection approach early in our experience.

Patients with a large or paraesophageal hernia were significantly older compared to those with a small or no hernia [60.4 (10.7) vs. 51.5 (14), *p *< 0.0001]. There was also a higher percentage of women among those with large or paraesophageal hernia (70% vs. 56.7%, *p *= 0.037).

At a mean follow-up of 13.6 (10.4) months, the rate of outcome satisfaction was high and similar between the four groups (*p *= 0.72). This was also true for GERD–HRQL total score clinical improvement (*p *= 0.77), the rate of freedom from PPI (*p *= 0.96), and normalization of distal esophageal acid exposure (*p *= 0.21) (Table 2).

**Table 2:** Subjective and objective outcome measures 1 year after MSA

Measurement	*N* (%)	Baseline hiatal hernia status				*p* Value
		None *N* (%)	Small *N* (%)	Large *N* (%)	PEH *N* (%)	
Total	350 (100.0)	65 (18.6)	205 (58.6)	58 (16.6)	22 (6.2)	N/A
Satisfaction from surgery	277					
No	31 (11.2%)	6 (13.3%)	20 (12.1%)	4 (7.8%)	1 (6.2%)	0.73
Yes	246 (88.8%)	39 (86.7%)	145 (87.9%)	47 (92.2%)	15 (93.8%)	
GERD–HRQL total score clinical improvement	280					
No	58 (20.7%)	9 (20.9%)	38 (22.2%)	9 (18.0%)	2 (12.5%)	0.77
Yes	222 (79.3%)	34 (79.1%)	133 (77.8%)	41 (82.0%)	14 (87.5%)	
Normalization of acid exposure	193					
DeMeester score < 14.7	143 (74.1%)	27 (71.1%)	88 (79.3%)	21 (65.6%)	7 (58.3%)	0.21
Freedom from PPI use	282					
Yes	259 (91.8%)	41 (93.2%)	156 (91.8%)	47 (90.4%)	15 (93.8%)	0.96
No	23 (8.2%)	3 (6.8%)	14 (8.2%)	5 (9.6%)	1 (6.2%)	

### Perioperative complications and hospital stay

Ninety percent of the patients with no hernia or a small hernia were discharged home on the same day of surgery. In contrast 76% of those with a large or paraoesophageal hernia were discharged home on the same day (Table 3). The reasons for overnight stay were: poor post-op pain control (*n *= 2), CO_2_ retention and need for re-intubation (*n *= 1), significant post-op nausea (*n *= 3), need for supplemental oxygen (*n *= 6) and lethargy (*n *= 2). In the remaining 13 patients, the overnight observation was due to patient request, presence of comorbidities or advanced age.

**Table 3:** Hospital stay and complication and readmission rates (within 90 days)

Measurement	*N* (%)	Baseline hiatal hernia status				*p* Value
		None *N* (%)	Small *N* (%)	Large *N* (%)	PEH *N* (%)	
Total	350 (100.0%)	65 (18.6%)	205 (58.6%)	58 (16.6%)	22 (6.2%)	
Hospitalization						
Same day discharge	323 (92.3%)	61 (93.9%)	196 (95.6%)	49 (84.5%)	17 (77.3%)	*p *= 0.002
≥ One day hospital stay	27 (7.7%)	4 (6.1%)	9 (4.4%)	9 (15.5%)	5 (22.7%)	
Readmission within 90 days	19 (5.4%)	0 (0.0%)	14 (6.8%)	2 (3.5%)	3 (13.7%)	*p *= 0.049
Major complications^a^	2 (0.6%)	0 (0.0%)	0 (0.0%)	2 (3.4%)	0 (0.0%)	
Minor complications^b^	37 (10.6%)	2 (3.1%)	23 (11.2%)	6 (10.4%)	6 (27.3%)	
Overall complications	39 (11.1%)	2 (3.1%)	23 (11.2%)	8 (13.8%)	6 (27.3%)	*p *= 0.015

^a^Major complications include CO_2_ retention requiring re-intubation (*n *= 1) and mediastinal abscess requiring drainage and IV antibiotic (*n *= 1)

^b^Minor complications include poor postoperative pain control (*n *= 2), significant nausea during immediate postoperative period (*n *= 3), hypoxia requiring supplemental oxygenation (*n *= 6), lethargy (*n *= 2), abdominal pain requiring further evaluation (*n *= 5), persistent nausea and vomiting *n *= (8), abdominal wall hematoma at gastric pacer insertion site (*n *= 1), DVT (*n *= 1), urinary retention (*n *= 1), and dyspnea requiring further work-up (*n *= 2)

A total of 19 patients required readmission within 90 days after surgery. One patient was readmitted three times; this patient also had implantation of a gastric stimulator at the time of MSA. This patient required two admissions for persistent nausea and vomiting and one admission for hematoma at the site of gastric stimulator within the anterior abdominal wall. Two patients required two readmissions and the remaining patients were readmitted only once after MSA (Table 3).

### Postoperative dysphagia and need for intervention

There was an improvement in the overall prevalence of dysphagia when compared to baseline (11.7 vs. 35%, *p *< 0.001). The rate of postoperative dysphagia was similar among the four groups (*p *= 0.33, Table 4) and there was no difference between the need for dilation among groups (*p *= 0.13). The need for device removal (5% overall) was similar between the four groups (*p *= 0.28). All the removals were for persistent dysphagia or esophageal spasm unresponsive to endoscopic dilation. There were no device erosions in this series.

**Table 4:** Rate of dysphagia, need for dilation or device removal

Measurement	*N* (%)	Baseline hiatal hernia status				*p* Value
		None *N* (%)	Small *N* (%)	Large *N* (%)	PEH *N* (%)	
Dysphagia	41 (15.3%)	6 (15.8%)	31(15.7%)	3 (5.8%)	1 (5.9%)	0.08
Need for endoscopic dilation	82 (23.4%)	13 (20.0%)	54 (26.3%)	14 (24.1%)	1 (4.5%)	0.12
Device removal	18 (5.1%)	4 (6.1%)	13 (6.4%)	1 (1.7%)	0 (0.0%)	0.28

### Objective follow-up and hernia recurrence

**Fig. 2:**
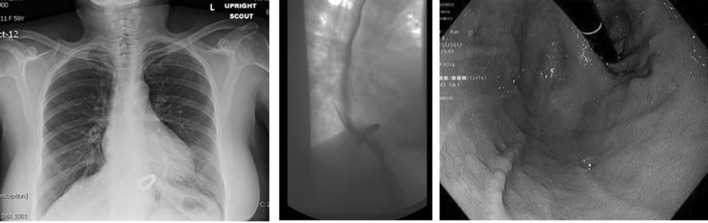
Radiologic and endoscopic appearance of a type I-b hernia recurrence after MSA with minimal dissection. This patient underwent endoscopic balloon dilation of the GEJ and a short course of steroid with resolution of her symptoms

**Fig. 3:**
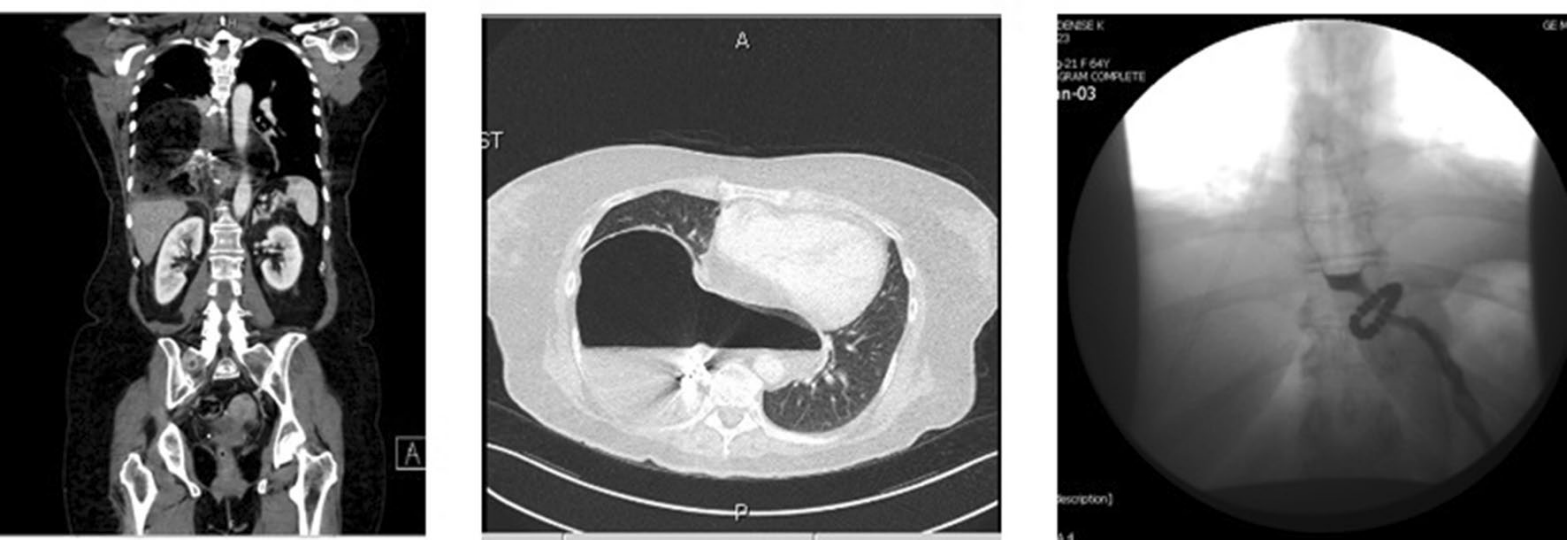
CT scan of a patient with type III recurrence. Patient underwent reoperation for repair of PEH and LINX device was left in position. The postoperative esophagram after reoperation demonstrates an appropriately positioned LINX® and no herniation

**Fig. 4:**
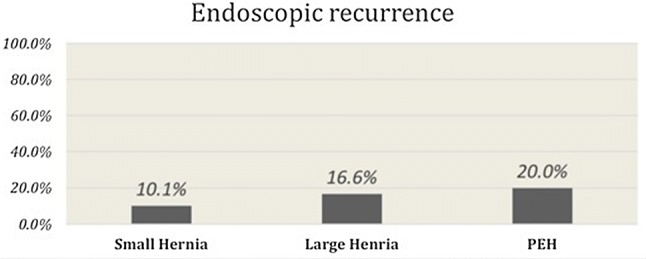
Incidence (%) of endoscopic hiatal hernia recurrence across the groups

A total of 240 of 350 (69%) patients underwent upper endoscopy on their annual follow-up visit. Based on the observed patterns of HH recurrence and insights obtained from reoperation, we have established a classification system and proposed management strategy (Table 5). Radiologic and endoscopic appearance of a type I-b hernia recurrence is shown in Fig. 2 and radiologic images of a patient with type III recurrence are shown in Fig. 3. The rate of HH recurrence on upper endoscopy was 10% (*n *= 24) in all groups combined. Of these, 20 (83.3%) had a small hernia, 1 (4.2%) had a large hernia and 3 (12.5%) had a paraesophageal hernia. Recurrence rate increased in a stepwise fashion with an increase in preoperative HH size (0%, 10.1%, 16.6% and 20%, *p *= 0.032, Fig. 4). Patients with a minimal dissection had a higher hiatal hernia recurrence rate compared to those with a full dissection (21% vs. 7.9%, *p *= 0.033).

**Table 5:** Classification of symptomatic recurrence of hiatal hernia after MSA and proposed management

Type I-a	HH recurrence with properly placed device in relation GEJ	Repair hernia and leave the device in position
Type I-b	HH recurrence with position of device located too proximal to GEJ	Endoscopic dilation under fluoroscopy and short course of steroid (if recurrence is small and occurs within 3 months from surgery) If large symptomatic recurrence, go directly to HH repair and replace device
Type I-c	HH recurrence with location of device on cardia or proximal stomach	If normal or mildly reduced LES pressure and/or length on preoperative manometry, repair HH and remove device without further intervention If absent or markedly reduced LES pressure and/or length on preoperative manometry, repair hernia and replace device or perform fundoplication
Type II^a^	Paraesophageal re-herniation with GEJ in intraabdominal location	Device in proper position at GEJ: Repair hernia ± biologic mesh and leave device in place Device in wrong location (too proximal or too distal) in relation to GEJ: Repair hernia ± biologic mesh and replace device or perform fundoplication
Type III^a^	Paraesophageal re-herniation with stomach and GEJ located intrathoracically	Device in proper position at GEJ: Repair hernia ± biologic mesh and leave device in place Device in wrong location in relation to GEJ (too proximal or too distal): Repair hernia ± biologic mesh and replace device or perform fundoplication

^a^Type II and III can also be divided into three subcategories and as described above the inappropriate position of the device in relation to GEJ will require device replacement

Of 24 patients found to have hiatal hernia recurrence on endoscopy, 7 required reoperation (Table 6). Patient with minimal dissection was more likely to require reoperation compared to those with a full dissection (10.5% vs. 1.5%, *p *= 0.0133). Factors associated with recurrence of hernia in these 7 patients were increase in body mass index after MSA, participation in activities or conditions that increase intrabdominal pressure, and the minimal dissection approach to MSA. We observed that the majority of patients with symptomatic recurrence presented with new onset dysphagia with or without upper abdominal pain; and 2 patients that originally presented with cough had return of this symptom with recurrence.

**Table 6:** Characteristics and intraoperative findings on the revisional surgery of patients with recurrent hernia

	Age/sex	Baseline BMI vs. recurrence BMI	Activities or conditions increaseing IAP (Y/N)	Baseline HH type	Minimal dissection approach (Y/N)	Symptom resolution after primary MSA (Y/N)	Recurrent symptoms (Y/N)	Time to recurrence (months)	Revisional surgery findings	Revisional surgery performed
1	65/F	34.1 vs. 36	N	I (1 cm)	Y	Y	Y (new onset abdominal pain)	18	Type I-c: LINX® and proximal stomach herniated into chest and LINX® located on the gastric cardia	Hernia reduction, full dissection with cruralplasty, device removed and replaced
2	23/M	31 vs. 28	Y (IBS)	I(1 cm)	Y	Y	Y (new onset dysphagia)	2	Type I-c: LINX® herniated into hiatal opening and located on the gastric cardia	Hernia reduction, full dissection with cruralplasty, device removed and replaced
3	64/M	30 vs. 33.4	Y (weight lifting)	I (3 cm)	N (full dissection)	Y	Y (recurrent cough)	7	Type II: Posterior herniation of gastric fundus with LINX® device in proper location at GEJ and in intraabdominal location	Reduction of hernia, further esophageal mobilization and repeat cruralplasty
4	64/F	29.6 vs. 30	N	III (entire stomach)	N (full dissection)	Y	Y (new onset abdominal pain and dysphagia)	3	Type III: recurrent PEH with LINX® in proper location at level of GEJ but herniated intrathoracically; separation of hiatal closure	Reduction of hernia and crural closure with device left in place
5	73/M	25.6 vs. 28	Y (cough)	I (2 cm)	Y	Y	Y (recurrent cough)	42	Type I-a: LINX® herniated but in proper position at GEJ; LINX® also herniated intrathoracically	Reduction of hernia, full dissection with cruralplasty; LINX left in place
6	61/M	26 vs. 25.6	Y (exercise with bearing down at 2 weeks)	I (1 cm)	N (full dissection)	Y	Y (new onset dysphagia)	< 1	Type I-c: LINX® and proximal stomach herniated into chest and LINX® located on the gastric cardia	Reduction of hernia, further esophageal mobilization and repeat cruralplasty with biologic mesh; LINX removed and replaced
7	47/M	37.1 vs. 38	Y	I (2 cm)	Y	Y	Y (Heartburn and regurgitation)	24	Type III: PEH with LINX® in proper location at level of GEJ but herniated into chest; separation of hiatal closure	Hernia reduction, full dissection with repeat cruralplasty; LINX® left in position

Of the patients that returned for high-resolution manometry (*n *= 95) at 1-year following MSA, there was no difference in esophageal function (peristalsis or pressure) when compared to preoperative values. Of note, with surgical correction of PEH and MSA, there was marked improvement in preoperative incomplete bolus clearance in this group (Table 7).

**Table 7:** Preoperative and postoperative esophageal body manometric characteristic across the four groups

Measurement	Baseline hiatal hernia status				*p* Value
	None Mean (SD)	Small Mean (SD)	Large Mean (SD)	PEH Mean (SD)	
Mean wave amplitude (mmHg)					
Preoperative value, mean (SD)	101 (48)	85 (38)	88 (47)	85 (34)	0.45
Postoperative value mean (SD)	122 (54)	100 (38)	93 (39)	83 (37)	0.24
Mean DCI (mmHg.s.cm)					
Preoperative value, mean (SD)	2368 (1971)	1881 (1380)	2201 (2432)	1332 (708)	0.24
Postoperative value mean (SD)	3452 (2379)	2231 (1313)	2848 (3009)	1645 (919)	0.32
% Peristaltic waves					
Preoperative value, mean (SD)	91 (17)	90 (18)	89 (18)	95 (9)	0.59
Postoperative value Mean (SD)	90 (15)	88 (19)	88 (17)	88 (13)	0.97
% Incomplete bolus clearance					
Preoperative value, mean (SD)	16 (27)	22 (33)	32 (37)	43 (45)	0.007
Postoperative value mean (SD)	15 (22)	29 (36)	30 (36)	16 (21)	0.61

## Discussion

Reflux disease and its complications are the consequences of progressive anatomic and mechanical defects. Hiatal hernia plays an important role in this disease process and its repair in addition to fundoplication is of critical importance in every antireflux surgery [30]. At the outset, MSA was designed to support a partially defective LES and prevent its effacement with gastric distention and increases in intraabdominal pressure. The original intent of MSA was placement in the earlier stages of GERD (without hiatal derangement) to stop symptoms and prevent progression to a large hiatal hernia with severe bi-positional volume reflux.

As more MSA procedures were performed in early stage GERD patients, it became evident that many HH were subclinical and only discovered at the time surgery in the form of axial displacement and/or severe transverse hiatal widening. To perform only MSA in these patients without HH repair would deprive the barrier of its crural contribution and violate a fundamental tenant of antireflux surgery. It is for these reasons that the current trend in MSA is to couple this procedure with a full mediastinal esophageal dissection and cruroplasty regardless of HH status.

In the present study, we demonstrated that equivalent degrees of symptomatic improvement and freedom from PPI are achieved with MSA across the spectrum of HH type and size. These results are comparable to those obtained nationally in the feasibility, pivotal and single center experiences in highly selected patients with small or no HH. When MSA is used in the setting of HH repair, there is realignment of the extrinsic and intrinsic components of the antireflux barrier plus tightening of the former and augmentation of the latter, which enables both elements to effectively function as a single unit.

Two recent studies have shown encouraging results in the use of MSA coupled with repair of larger sized HH, and reported improved outcomes in those with ≥ 3 cm hernia [17, 18] compared to those with little or no HH who underwent the minimal dissection approach. These studies did not include patients with paraesophageal hernia [17] and also did not compare the objective surgical outcome across the spectrum from no HH to PEH [18]. This initial work set the stage for the surgical community to address the fundamental importance of the crural contribution in all forms of antireflux surgery including novel technologies such as MSA.

Surgical repair is indicated in patients with a symptomatic large or paraesophageal hernia. An antireflux procedure is commonly added to the repair of these large hernias. This is due to the high likelihood of symptomatic postoperative reflux and theoretically, to minimize the chance of re-herniation. However, studies have demonstrated that hiatal hernia repair is associated with a high rate of recurrence irrespective of size and type of hernia, technique of repair and use of mesh. This rate is reported to be as high as 67% [27–32]. Stirling and Orringer demonstrated that 72% of patients who required reoperation for recurrent reflux disease were found to have failure primarily due to breakdown of the crural repair [33].

By contrast, studies on MSA patients have reported a lower rate of hiatal hernia recurrence when compared to the rates reported with fundoplication [17]. Similarly, we observed an overall recurrence rate of 10% and only 7 (2.9%) patients were symptomatic and required reoperation within the 1-year follow-up period. It is likely that based on the favorable late follow-up questionnaire data, the majority of patients that did not present for 1-year follow-up objective testing were asymptomatic and without recurrence; this would further reduce the HH recurrence rate to below 2.9% in this series.

Recurrence rate increased in a stepwise fashion with an increase in preoperative HH size; however, it is interesting to note that most of the patients that required reoperation had a smaller size type I hernia and underwent the minimal dissection approach. Although the patients who underwent minimal dissection had a lower rate of hiatal hernia on their preoperative EGD, they had a higher rate of hiatal hernia recurrence requiring reoperation compared to those with a full dissection (10.5% vs. 1.9%, *p *= 0.013). Therefore, the minimal dissection approach to MSA should be abandoned.

Two explanations for the lower rate of recurrence after MSA have been proposed [17]. One is the preserved ability to belch after MSA thereby enabling consistent gastric decompression. This ability is lost after a Nissen fundoplication resulting in elevation in intragastric pressure, which imposes external force on the hiatus and fundoplication during the healing process and beyond. The second explanation is that the titanium device may provide a beneficial inflammatory response with subsequent scarring, thereby solidifying the hiatal closure and securing the GEJ in an intraabdominal location.

Our re-operative experience on patients after MSA supports this supposition, and we observed robust scarring around the device, GEJ, and crura during removal for dysphagia. Examination of tissues adjacent to orthopedic titanium plates has shown a chronic and sustained inflammatory response with abundant macrophages and fibroblasts [34]. This reaction may represent a benefit of MSA over Nissen fundoplication in terms of prevention of migration as well as reinforcement of the hiatal closure.

Our results establish that the application of MSA in the HH patient is safe with overall and major complication rates of 11% and 0.6%, respectively. However, overall complications, length of stay, and 90-day readmission rates were higher in patients with large and paraesophageal hernia. In comparison with other groups, large and paraesophageal hernia groups of patients were older and had more co-morbid conditions than patients with small or no hernia. Using the National Surgical Quality Improvement Program (NSQIP) database, Molena and colleagues found advanced age to be associated with increased rates of 30-day morbidity, mortality, and length of stay in patients undergoing antireflux surgery [35].

We studied the impact of hiatal hernia in the largest series of patients with objective follow-up data. This study however is limited by its retrospective nature and lack of objective follow-up in 100% of the patients. Further, the surgeries were done in a high-volume center by surgeons with a focused foregut practice and in patients who underwent a detailed preoperative evaluation. It is possible that these results may not reflect broader clinical practice.

## Conclusion

In the largest series of MSA implantation, we demonstrate that the excellent outcomes and high degree of satisfaction after MSA are independent of the presence or size of HH. Despite higher rates of hernia recurrence in large HH and PEH patients, the rates of postoperative endoscopic intervention, and device removal is similar to those with no or small HH. Patients with a minimal dissection have a higher rate of recurrent HH and are more likely to require reoperation compared to those with a full dissection. Therefore, the minimal dissection approach to MSA should be abandoned.
